# Comparing oral case presentation formats on internal medicine inpatient rounds: a survey study

**DOI:** 10.1186/s12909-023-04292-3

**Published:** 2023-05-24

**Authors:** Brendan Appold, Sanjay Saint, David Ratz, Ashwin Gupta

**Affiliations:** 1 University of Michigan Medical School, Ann Arbor, MI USA; 2 VA Ann Arbor Healthcare System, 2215 Fuller Road, Ann Arbor, MI 48105 USA

**Keywords:** EAP, Medical education, Oral case presentations, Rounding, SOAP

## Abstract

**Background:**

Oral case presentations – structured verbal reports of clinical cases – are fundamental to patient care and learner education. Despite their continued importance in a modernized medical landscape, their structure has remained largely unchanged since the 1960s, based on the traditional Subjective, Objective, Assessment, Plan (SOAP) format developed for medical records. We developed a problem-based alternative known as Events, Assessment, Plan (EAP) to understand the perceived efficacy of EAP compared to SOAP among learners.

**Methods:**

We surveyed (Qualtrics, via email) all third- and fourth-year medical students and internal medicine residents at a large, academic, tertiary care hospital and associated Veterans Affairs medical center. The primary outcome was trainee preference in oral case presentation format. The secondary outcome was comparing EAP and SOAP on 10 functionality domains assessed via a 5-point Likert scale. We used descriptive statistics (proportion and mean) to describe the results.

**Results:**

The response rate was 21% (118/563). Of the 59 respondents with exposure to both the EAP and SOAP formats, 69% (*n* = 41) preferred the EAP format as compared to 19% (*n* = 11) who preferred SOAP (*p* < 0.001). EAP outperformed SOAP in 8 out of 10 of the domains assessed, including advancing patient care, learning from patients, and time efficiency.

**Conclusions:**

Our findings suggest that trainees prefer the EAP format over SOAP and that EAP may facilitate clearer and more efficient communication on rounds, which in turn may enhance patient care and learner education. A broader, multi-center study of the EAP oral case presentation will help to better understand preferences, outcomes, and barriers to implementation.

**Supplementary Information:**

The online version contains supplementary material available at 10.1186/s12909-023-04292-3.

## Background

Excellent inter-physician communication is fundamental to both providing high-quality patient care and promoting learner education [1], and has been recognized as an important educational goal by the Clerkship Directors in Internal Medicine, the Association of American Medical Colleges, and the Accreditation Council for Graduate Medical Education [2]. Oral case presentations, structured verbal reports of clinical cases [3], have been referred to as the “currency with which clinicians communicate” [4]. Oral case presentations are a key element of experiential learning in clinical medicine, requiring learners to synthesize, assess, and convey pertinent patient information and to formulate care plans. Furthermore, oral case presentations allow supervising clinicians to identify gaps in knowledge or clinical reasoning and enable team members to learn from one another. Despite modernization in much of medicine, oral case presentation formats have remained largely unchanged, based on the traditional Subjective, Objective, Assessment, Plan (SOAP) format developed by Dr. Lawrence Weed in his Problem Oriented Medical Record in 1968 [5].

Given that the goals of a medical record are different than those of oral case presentations, it should not be assumed that they should share the same format. While Dr. Weed sought to make the medical record as “complete as possible,” [6] internal medicine education leaders have expressed desire for oral case presentations that are succinct, with an emphasis on select relevant details [2]. Using a common SOAP format between the medical record and oral case presentations risks conflating the distinct goals for each of these communication methods. Indeed, in studying how learners gain oral case presentation skills, Haber and Lingard [7] found differences in understanding of the fundamental purpose of oral case presentations between medical students and experienced physicians. While students believed the purpose of oral case presentations was to organize the large amount of data they collected about their patients, experienced physicians saw oral case presentations as a method of telling a story to make an argument for a particular conclusion [7].

In accordance with Dr. Weed’s “problem-oriented approach to data organization,” [6] but with an eye toward optimizing for oral case presentations, we developed an alternative to SOAP known as the Events, Assessment, Plan (EAP) format. The EAP format is used for patients who are already known to the inpatient team, and may also be utilized for newly admitted patients for whom the attending physician already has context (e.g., via handoff or review of an admission note). As the EAP approach is utilized by a subset of attending physicians at our academic hospital, we sought to understand the perceived effectiveness of the EAP format in comparison to the traditional SOAP format among learners (i.e., medical students and resident physicians).

## Methods

### EAP format

**Fig. 1:**
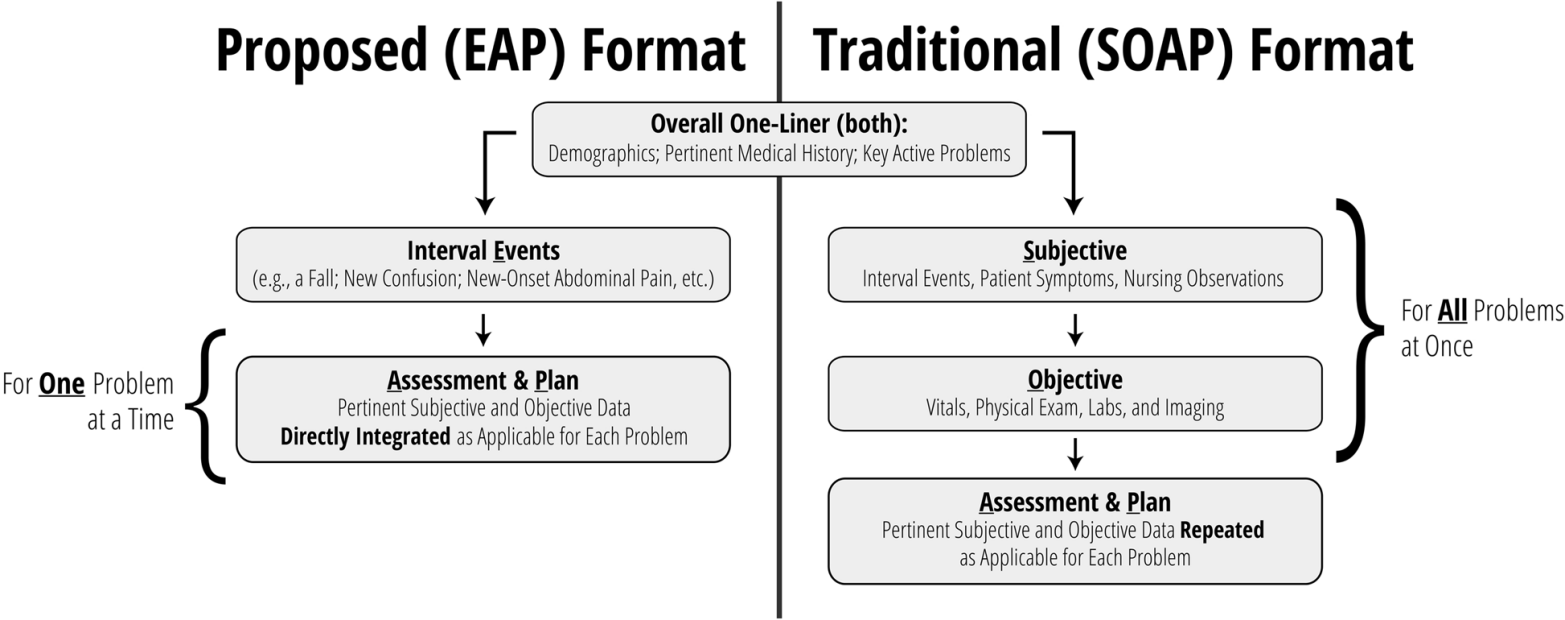
Overview: comparing EAP to SOAP

EAP is a problem-based format used at the discretion of the attending physician. In line with suggested best practices [8], the EAP structure aims to facilitate transmission of data integrated within the context of clinical problem solving. In this format, significant interval events are discussed first (e.g., a fall, new-onset abdominal pain), followed by a prioritized assessment and plan for each relevant active problem. Subjective and objective findings are integrated into the assessment and plan as relevant to a particular problem. This integration of subjective and objective findings by problem is distinct from SOAP, where subjective and objective findings are presented separately as their own sections, with each section often containing information that is relevant to several problems (Fig. 1, Additional file 1: Appendix A).

### Settings and participants

We surveyed third- and fourth-year medical students, and first- through fourth-year internal medicine and internal medicine-pediatrics residents, caring for patients at a large, academic, tertiary care hospital and an affiliated Veterans Affairs medical center. Internal medicine is a 12-week core clerkship for all medical students in their second year, with 8 weeks spent on the inpatient wards. All student participants had completed their internal medicine clerkship rotation at the time of the survey. We did not conduct a sample size calculation at the outset of this study.

### Data collection methods and processes

An anonymous, electronic survey (Qualtrics, Provo, UT) was created to assess student and resident experience with and preference between EAP and SOAP oral case presentation formats during inpatient internal medicine rounds (Additional file 2: Appendix B). Ten domains were assessed via 5-point Likert scale (1 [strongly disagree] to 5 [strongly agree]), including the ability of the format to incorporate the patient’s subjective experience, the extent to which the format encouraged distillation and integration of information, the extent to which the format focused on the assessment and plan, the format’s ability to help trainees learn from their own patients and those of their peers, time efficiency, and ease of use. Duration of exposure to each format was also assessed, as were basic demographic data for the purposes of understanding outcome differences among respondents (e.g., students versus residents). For those who had experienced both formats, preference between formats was recorded as a binary choice. Participants additionally had the opportunity to provide explanation via free text. For participants with experience in both formats, the order of evaluation of EAP and SOAP formats were randomized by participant. For questions comparing EAP and SOAP formats directly, choice order was randomized.

The survey was distributed via official medical school email in October 2021 and was available to be completed for 20 days. Email reminders were distributed approximately one week after distribution and again 48 h prior to survey conclusion.

### Outcomes

The primary outcome was trainee preference in oral case presentation format. Secondary outcomes included comparison between EAP and SOAP on content inclusion/focus, data integration, learning, time efficiency, and ease of use.

### Statistical analyses

Descriptive statistics were used to describe the results (proportion and mean). For comparative analysis between EAP and SOAP, responses from respondents who had experience with both formats were compared using the Wilcoxon Signed Rank Test to evaluate differences. All statistical analyses were done using SAS V9.4 (SAS Institute, Cary, NC). We considered *p* < 0.05 to be statistically significant.

## Results

The overall response rate was 21% (118/563). The response rate was 14% (*n* = 62/441) among medical students and 46% (*n* = 56/122) among residents. Respondents were 61% (*n* = 72) female. A total of 98% (*n* = 116) and 52% (*n* = 61) of respondents reported experience with SOAP and EAP formats, respectively. Among medical students, 60% (*n* = 37) reported experience with SOAP only while 39% (*n* = 24) had experience with both formats. Among residents, 36% (*n* = 20) and 63% (*n* = 35) had experience with SOAP only and both formats, respectively (Table 1). Most students (93%) and residents (96%) reported > 8 weeks of exposure to the SOAP format. Duration of exposure to the EAP format varied (0 to 2 weeks [32% of students, 17% of residents], 2 to 4 weeks [36% of students, 47% of residents], 4 to 8 weeks [16% of students, 25% of residents], and > 8 weeks [16% of students, 11% of residents]).

**Table 1:** Quantifying trainees who only experienced SOAP versus those who experienced both formats

Trainee Group	% SOAP Only	% EAP and SOAP
Medical Students	60 (*n* = 37)	39 (*n* = 24)
Residents	36 (*n* = 20)	63 (*n* = 35)

Of the 59 respondents with exposure to both the SOAP and EAP formats, 69% (*n* = 41) preferred the EAP format as compared to 19% (*n* = 11) preferring SOAP (*p* < 0.001). The remainder (*n* = 7, 12%) indicated either no preference between formats or indicated another preference. Among residents, 66% (*n* = 23) favored EAP, whereas 20% (*n* = 7) and 14% (*n* = 5) preferred SOAP or had no preference, respectively (*p* < 0.001). Among students, 75% (*n* = 18) favored EAP, whereas 17% (*n* = 4) and 8% (*n* = 2) favored SOAP or had no preference, respectively (*p* < 0.001).

Likert scale ratings for domains assessed by trainees who had experience in either format are shown in Table 2. In general, scores for each domain were higher for EAP than SOAP, with the exception of perceived ease of use among students. Among those with experience using both formats, EAP outperformed SOAP most prominently in time efficiency (mean 4.39 vs 2.59, *p* < 0.001) and encouragement to: focus on assessment and plan (4.64 vs 3.05, *p* < 0.001), distill pertinent information (4.63 vs 3.17, *p* < 0.001), and integrate data (4.58 vs 3.31, *p* < 0.001) (Table 3). Respondents also ranked EAP higher in its effectiveness at advancing patient care (4.31 vs 3.71, *p* < 0.001), its capacity to convey one’s thinking (4.53 vs 3.95, *p* < 0.001), and its ability to facilitate learning from peers (4.10 vs 3.58, *p* < 0.001) and one’s own patients (4.24 vs 3.78, *p* = 0.003). There were no significant differences in the amount of time allotted for discussing the patient’s subjective experience or in ease of use.

**Table 2:** Domain ratings for the EAP and SOAP formats for all respondents with exposure to either format^a^

	**Students**		**Residents**	
**Assessment Domain**	**EAP** **(*n* = 25)**	**SOAP** **(*n* = 61)**	**EAP** **(*n* = 36)**	**SOAP** **(*n* = 55)**
Allowed you to adequately convey your thought process	4.48	3.97	4.56	3.78
Allowed adequate time for discussion of the patient’s subjective experience	4.04	3.92	4.36	3.87
Encouraged you to distill pertinent information in your presentation	4.68	3.63	4.61	3.33
Encouraged you to integrate information from the history, exam, and studies in developing an assessment and plan	4.68	3.63	4.53	3.53
Encouraged you to focus on your assessment and plan	4.64	3.50	4.67	3.13
Helped you learn from your own patients	4.28	3.95	4.25	3.71
Helped you learn from your peers	4.16	3.70	4.11	3.58
Is effective in advancing patient care	4.44	3.83	4.25	3.63
Is time-efficient	4.44	2.93	4.36	2.64
Is easy to use	3.88	4.02	4.03	3.80

^a^ Mean scores to the prompt: “The ‘___’ presentation format…”

(1 = strongly disagree, 2 = disagree, 3 = neither disagree nor agree, 4 = agree, 5 = strongly agree)

**Table 3:** EAP vs SOAP head-to-head for all respondents who experienced both formats^a^

	**Students (*n* = 24)**			**Residents (*n* = 35)**			**Total (*n* = 59)**		
**Assessment Domain**	**EAP**	**SOAP**	***P*-value**	**EAP**	**SOAP**	***P*-value**	**EAP**	**SOAP**	***P*-value**
Allowed you to adequately convey your thought process	4.50	4.04	0.07	4.54	3.89	**0.003**	4.53	3.95	** < .001**
Allowed adequate time for discussion of the patient’s subjective experience	4.04	4.13	0.69	4.34	3.89	**0.02**	4.22	3.98	0.17
Encouraged you to distill pertinent information in your presentation	4.67	3.13	** < .001**	4.60	3.20	** < .001**	4.63	3.17	** < .001**
Encouraged you to integrate information from the history, exam, and studies in developing an assessment and plan	4.67	3.13	** < .001**	4.51	3.43	** < .001**	4.58	3.31	** < .001**
Encouraged you to focus on your assessment and plan	4.63	3.17	** < .001**	4.66	2.97	** < .001**	4.64	3.05	** < .001**
Helped you learn from your own patients	4.25	3.88	0.09	4.23	3.71	**0.02**	4.24	3.78	**0.003**
Helped you learn from your peers	4.13	3.58	**0.01**	4.09	3.57	**0.01**	4.10	3.58	** < .001**
Is effective in advancing patient care	4.42	3.83	**0.02**	4.23	3.63	**0.01**	4.31	3.71	** < .001**
Is time-efficient	4.46	2.58	** < .001**	4.34	2.60	** < .001**	4.39	2.59	** < .001**
Is easy to use	3.88	4.04	0.55	4.00	3.82	0.62	3.95	3.91	0.98

^a^ Mean scores to the prompt: “The ‘___’ presentation format…”

(1 = strongly disagree, 2 = disagree, 3 = neither disagree nor agree, 4 = agree, 5 = strongly agree)

Evaluation of trainee free text responses regarding oral case presentation preference revealed several general themes (Table 4). First, respondents generally felt that EAP was more time efficient and less repetitive, allowing for additional time to be spent discussing pertinent patient care decisions. Second, several respondents indicated that EAP aligns well with how trainees consider problems naturally (as a single problem in completion). Finally, respondents generally believed that EAP allowed learners to effectively communicate their thinking and demonstrate their knowledge. Those preferring SOAP most often cited format familiarity and the difficulty in switching between formats in describing their preference, though some also believed SOAP was more effective in describing a patient’s current status.

**Table 4:** Themes related to format preference

Theme	Representative Quotations
EAP is time efficient and less repetitive, allowing for discussion of critical components of patient care	*much more efficient and avoids repetition… allow[ing] us to spend more time talking with patients instead of about [them] (Student)* *faster and incorporates pertinent information where it is needed (Resident)* *provides an opportunity for students to consolidate their understanding of a patient’s current condition and the plan for moving them forward (Student)* *allows for the majority of our time to be spent discussing the component that is most important: the assessment and plan (Student)* *more concise and only includes relevant information (Resident)*
EAP follows a more natural thought process	*follows logical thought processes, the way I actually think about the patient and synthesize their data (Resident)* *ideas flow more naturally, and connections are better highlighted (Student)* *integrates your information with your assessment and plan to provide a cohesive story of the patient’s current presentation (Resident)* *allows information to be presented in context… where it is most relevant (Resident)* *better fits how attendings and experienced trainees more commonly communicate with one another (Resident)*
EAP allows for communication of thinking and demonstration of knowledge	*allows the student to show off their medical knowledge by correctly grouping data (Student)* *a better way for students to show what they are thinking and what they know (Student)* *encouraged intentional thought about subjective/objective data and how it affects each problem (Student)* *forces you to think critically about why you’re doing the things you’re doing for the patient, better focus on the assessment and plan (Student)* *allows me to demonstrate my clinical judgement (Resident)*
SOAP is more familiar and switching between formats can be difficult	*more universally used on other services so we are more accustomed to it.. following one template is easier than trying to switch how you present depending on each attending (Student)* *the much more common format and it's what most medical students are taught in school… when we [ask] medical students to present in an entirely different format they seem to get hung up on making sure things are in this unfamiliar format and it takes away from them practicing the important clinical skills that come from giving strong oral presentations… presenting on rounds is where medical students do some of their most important learning, and adding logistical confusion to that process only seems to take away from their opportunities to both learn and build confidence in their clinical reasoning (Resident)* *this is the method that I am most comfortable with (Resident)* *I find the SOAP format easier to use because that is what I have traditionally been taught… I see merits to the EAP approach, however (Resident)* *I prefer SOAP mostly because I'm far more familiar with it… I used EAP for a few days with a new attending that came on and it was kind of confusing and I was not sure what exactly the difference was and what qualifies as an "event"… if I had more experience with EAP, it may be my preferred one (Student)*
SOAP helps illuminate the patient’s current status	*allows for deep thought about each piece of information [with regards to] the patient's current status (Student)* *makes sure people know what is happening (Resident)* *hearing the interval, subjective, and objective information first allows me to paint a picture of the current situation prior to hearing the assessment and plan (Resident)*

## Discussion

Our single site survey comparing 2 oral case presentation formats revealed a preference among respondents for EAP over SOAP for those medical students and internal medicine residents who had experience with both formats. Furthermore, EAP outperformed SOAP in 8 out of 10 of the functionality domains assessed, including areas such as advancing patient care, learning from patients, and, particularly, time efficiency. Such a constellation of findings implies that EAP may not only be a more effective means to accomplish the key goals of oral case presentations, but it may also provide an opportunity to save time in the process. In line with SOAP’s current de facto status as an oral case presentation format, almost all respondents reported exposure to the SOAP format. Still, indicative of EAP’s growing presence at our academic system, more than one third of medical students and more than one half of residents also reported having experience with the EAP format.

While limited data exist that compare alternative oral case presentations to SOAP on inpatient medicine rounds, such alternatives have been previously trialed in other clinical venues. One such format, the multiple mini-SOAP, developed for complex outpatient visits, encourages each problem to be addressed “in its entirety” before presenting subsequent problems, and emphasizes prioritization by problem pertinency [9]. The creators suggest that this approach encourages more active trainee participation in formulating the assessment and plan for each problem, by helping the trainee to avoid getting lost in an “undifferentiated jumble of problems and possibilities” [9] that accumulate when multiple problems are presented all at once. On the receiving end, the multiple mini-SOAP enables faculty to assess student understanding of specific clinical problems one at a time and facilitates focused teaching accordingly.

Another approach has been assessed in the emergency department. Specifically, Maddow and colleagues explored assessment-oriented oral case presentations to increase efficiency in communication between residents and faculty at the University of Chicago [10]. In the assessment-oriented format, instead of being presented in a stylized order, pertinent information was integrated into the analysis. The authors found that assessment-oriented oral case presentations were about 40% faster than traditional presentations without significant differences in case presentation effectiveness.

Prior to our study, the nature of the format for inpatient medicine oral case presentations had thus far escaped scrutiny. This is despite the fact that oral case presentations are time (and therefore resource) intensive, and that they play an integral role in patient care and learner education. Our study demonstrates that learners favor the EAP format, which has the potential to increase both the effectiveness and efficiency of rounding.

Still, it should be noted that a transition to EAP does present challenges. Implementing this problem-based presentation format requires a conscious effort to ensure a continued holistic approach to patient care: active problems should be defined and addressed in accordance with patient preferences, and the patient’s subjective experience should be meaningfully incorporated into the assessment and plan for each problem. During initial implementation, attending physicians and learners must internalize this new format, often through trial and error.

From there, on an ongoing basis, EAP may require more upfront preparation by attending physicians as compared to SOAP. While chart review by attendings in advance of rounding is useful regardless of the format utilized, this practice is especially important for the EAP format, where trainees are empowered to interpret and distill – rather than simply report a complete set of – information. Therefore, the attending physician must be aware of pertinent data prior to rounds to ensure that key information is not neglected. Specifically, attendings should pre-orient themselves with laboratory values, imaging, and other studies completed, and new suggestions from consultants. More extensive pre-work may be required if teams wish to employ the EAP format for newly admitted patients, as attending physicians must also familiarize themselves with a patient’s medical history and their current presentation prior to initial team rounds.

Our findings should be interpreted within the context of specific limitations. First, low response rates may have led to selection bias within our surveyed population. For instance, learners who desired change in the oral case presentation format may have been more motivated to engage with our survey. Second, there could be unmeasured confounding variables that could have skewed our results in favor of the EAP format. For example, attendings who utilized the EAP format may have been more likely to innovate in other ways to create a more positive experience for learners, which may have influenced the scoring of the oral case presentation format. Third, our findings were largely based on subjective experience. Objective measurement (e.g., duration of rounds, patient care outcomes) may lend additional credibility to our findings. Lastly, our study included only a single site, limiting our ability to generalize our findings.

Our study also had several strengths. Our learner participant pool was broad and included all third- and fourth-year medical students and all internal medicine residents at a major academic hospital. Participation was encouraged regardless of the nature of a participant’s prior exposure to different oral case presentation formats. Our survey was anonymous with randomization to mitigate order bias, and we focused our comparison analysis on those who had exposure to both the EAP and SOAP formats. We collected data to compare EAP with SOAP in 2 distinct ways: head-to-head preference and numeric ratings amongst key domains. Both of these methods demonstrated a significant preference for EAP among learners in aggregate, as well as for students and residents analyzed independently.

Our findings suggest a preference for the EAP format over SOAP, and that EAP may facilitate clearer and more efficient communication on rounds. These improvements may in turn enhance patient care and learner education. While our preliminary data are compelling, a broader, multi-center study of the EAP oral case presentation is necessary to better understand preferences, outcomes, and barriers to implementation. Further studies should seek to improve response rates, for the data to represent a larger proportion of trainees. One potential strategy to improve response rates among medical students and residents is to survey them directly at the end of each internal medicine clerkship period or rotation, respectively. Ultimately, EAP may prove to be a much-needed update to the “currency with which clinicians communicate.”

## Supplementary Information

**Additional file 1: Appendix A.** Exemplar Transcripts (EAP, SOAP).

**Additional file 2: Appendix B.** Survey Instrument.

## Data Availability

The data that support the findings of this study are available from the corresponding author, AG, upon reasonable request.
