# Supplementary material for: Comparing oral case presentation formats on internal medicine inpatient rounds: a survey study
Source: BMC Med Educ. 2023 May 24;23:377. doi: 10.1186/s12909-023-04292-3 (PMC10210329; doi:10.1186/s12909-023-04292-3)
Supplement: Supplementary file 1 — Additional file 1: Appendix A. Exemplar Transcripts (EAP, SOAP). [file 12909_2023_4292_MOESM1_ESM.docx]

*An Exemplar Transcript of an EAP Oral Case Presentation*

Mr. Smith is a 50-year-old man with a history of heart failure with reduced ejection fraction, stage 3 chronic kidney disease, and diabetes, who presented with concerns for viral pneumonia with possible superimposed bacterial infection, along with volume overload.

Interval Events: There were no events overnight.

Assessment and Plan by Problem.

*Problem 1: Pneumonia, bacterial on viral*

Mr. Smith was admitted three days ago with cough and shortness of breath, and a respiratory virus panel positive for rhinovirus-enterovirus; however, his chest x-ray also demonstrated a right lower lobe consolidation, and he had neutrophilia and an elevated procalcitonin all concerning for concurrent bacterial infection. His legionella and streptococcus urine antigens were negative, as was his MRSA nasal swab.

Vancomycin and piperacillin-tazobactam were discontinued yesterday, and he was transitioned to ampicillin-sulbactam. He remains afebrile and hemodynamically stable since the de-escalation of his antibiotics, and I believe it is reasonable to transition him to oral amoxicillin-clavulanic acid today to complete a 5-day course of antibiotics.

*Problem 2: Volume Overload*

We suspect decompensated heart failure based on nonadherence with his diuretic regimen for the week leading up to admission, presentation six pounds above his dry weight, shortness of breath, and hypoxia with a SpO2 of 86% on room air. Additionally, he had an elevated BNP and creatinine, and his transthoracic echocardiogram revealed a new ejection fraction of 20% from 40% previously.

*An Exemplar Transcript of an EAP Oral Case Presentation* (cont.)

*Problem 2: Volume Overload* (cont.)

Following aggressive diuresis over the past few days, Mr. Smith is approaching his dry weight, and his breathing and creatine levels have significantly improved. His peripheral edema is now 1+ and I appreciate no jugular venous distension. He will likely need a higher furosemide regimen given his decreased ejection fraction. We will diurese with furosemide 80mg once today and will consult cardiology for an ischemic evaluation given his newly reduced ejection fraction.

*An Exemplar Transcript of a SOAP Oral Case Presentation*

Mr. Smith is a 50-year-old man with a history of heart failure with reduced ejection fraction, stage 3 chronic kidney disease, and diabetes, who presented with concerns for viral pneumonia with possible superimposed bacterial infection, along with volume overload.

Subjective:

- There were no events overnight.
- Vancomycin and piperacillin-tazobactam were discontinued yesterday, and he was transitioned to ampicillin-sulbactam.
- Mr. Smith denies fevers/chills/nausea/vomiting.
- Mr. Smith’s oxygen was weaned yesterday.
- Mr. Smith believes his breathing has continued to improve.

Objective:

- Mr. Smith remained afebrile overnight with stable vitals. He continues to stat well on room air.
- Mr. Smith had a net output of 1.4 L yesterday with two doses of furosemide IV 80 mg, for a total net output of 2.7 L through this admission.
- Physical exam was notable for decreased edema from 2+ to 1+, and a lack of jugular venous distension.
- Blood cultures remain negative to date.
- His creatinine is down to 2.06 today from 2.15 from yesterday, and an overall high of 2.28; we are now approaching his baseline of 2.01.

*An Exemplar Transcript of a SOAP Oral Case Presentation* (cont.)

Assessment and Plan:

*Problem 1: Pneumonia, bacterial on viral*

Mr. Smith was admitted three days ago with cough and shortness of breath, and a respiratory virus panel positive for rhinovirus-enterovirus; however, his chest x-ray also demonstrated a right lower lobe consolidation, and he had neutrophilia and an elevated procalcitonin all concerning for concurrent bacterial infection. His legionella and streptococcus urine antigens were negative, as was his MRSA nasal swab.

Vancomycin and piperacillin-tazobactam were discontinued yesterday, and he was transitioned to ampicillin-sulbactam. He remains afebrile and hemodynamically stable since the de-escalation of his antibiotics, and I believe it is reasonable to transition him to oral amoxicillin-clavulanic acid today to complete a 5-day course of antibiotics.

*Problem 2: Volume Overload*

We suspect decompensated heart failure based on nonadherence with his diuretic regimen for the week leading up to admission, presentation six pounds above his dry weight, shortness of breath, and hypoxia with a SpO2 of 86% on room air. Additionally, he had an elevated BNP and creatinine, and his transthoracic echocardiogram revealed a new ejection fraction of 20% from 40% previously.

Following aggressive diuresis over the past few days, Mr. Smith is approaching his dry weight, and his breathing and creatine levels have significantly improved. His peripheral edema is now 1+ and I appreciate no jugular venous distension. He will likely need a higher furosemide regimen given his decreased ejection fraction. We will diurese with furosemide 80mg once today and will consult cardiology for an ischemic evaluation given his newly reduced ejection fraction.
