# Supplementary material for: Comparing oral case presentation formats on internal medicine inpatient rounds: a survey study
Source: BMC Med Educ. 2023 May 24;23:377. doi: 10.1186/s12909-023-04292-3 (PMC10210329; doi:10.1186/s12909-023-04292-3)
Supplement: Supplementary file 2 — Additional file 2: Appendix B. Survey Instrument. [file 12909_2023_4292_MOESM2_ESM.docx]

*Section 1: Introduction*

Oral case presentations are an integral part of internal medicine rounds. They provide an opportunity for team members to synchronize their thoughts, and thus are an important component of patient care. Additionally, they should facilitate experiential learning in the clinical sphere by a) requiring trainees to synthesize pertinent information to provide updates and formulate assessments and plans b) highlighting knowledge gaps that attendings can rectify and c) enabling team members to learn from one another. While the Subjective Objective Assessment Plan (SOAP) format is the dominant method used to structure oral case presentations, alternative approaches exist and are utilized at Michigan Medicine. One such approach is the problem-based Events Assessment Plan (EAP) format. In this format, subjective and objective findings are incorporated into the assessment and plan, rather than being presented separately. We want to understand your experience with different oral case presentation styles (even if you have only used the SOAP format) through this brief survey. Our goal is to optimize presentations to allow for better care of patients and better education of learners.

*Section 2: Consent*

This survey is for a study being done by researchers from the University of Michigan. We want to know what you think about different oral case presentation styles. Your answers will help researchers understand and improve presentations, the care of patients, and the education of learners.

Taking part in this survey is completely voluntary and should take less than 10 minutes. You may skip any question that you don’t want to answer. By completing this survey, you are agreeing to take part in this research study. Your answers will be kept confidential. We will not ask you for any information that could identify you, and your answers will not be shared outside the study team.

▢ I consent (1)

▢ I do not consent (2)

*Section 3: Format Exposure(s)*

Which presentation format(s) have you used on your internal medicine rotations?

▢ Subjective Objective Assessment Plan (SOAP) (1)

▢ Events Assessment Plan (EAP) (2)

▢ Both (3)

*Section 4: SOAP*

(for those who selected options 1 or 3 in Section 3)

Duration

How many weeks have you used the SOAP presentation format during your clinical training?

▢ 0 - 2 (1)

▢ 2 - 4 (2)

▢ 4 - 8 (3)

▢ 8+ (4)

Rating

The following questions ask about your views of the SOAP presentation format. For each of the statements below please indicate the extent of your agreement or disagreement by checking the appropriate box. (Questions listed below were presented in tabular format, with a 5-point Likert scale from 1 [strongly disagree] to 5 [strongly agree].)

The SOAP presentation format...

1. Allowed you to adequately convey your thought process
2. Allowed adequate time for discussion of the patient’s subjective experience
3. Encouraged you to distill pertinent information in your presentation
4. Encouraged you to integrate information from the history, exam, and studies in developing an assessment and plan
5. Encouraged you to focus on your assessment and plan
6. Helped you learn from your own patients
7. Helped you learn from your peers
8. Is effective in advancing patient care
9. Is time-efficient
10. Is easy to use

*Section 4: SOAP* (cont.)

Free Response 1

What do you like about the SOAP format?

Free Response 2

What do you dislike about the SOAP format?

*Section 5: EAP* (for those who selected options 2 or 3 in Section 3; for those who selected option 3 in Section 3, the order of presentation for Sections 4 and 5 was randomized)

Duration

How many weeks have you used the EAP presentation format during your clinical training?

▢ 0 - 2 (1)

▢ 2 - 4 (2)

▢ 4 - 8 (3)

▢ 8+ (4)

Rating

The following questions ask about your views of the EAP presentation format. For each of the statements below please indicate the extent of your agreement or disagreement by checking the appropriate box. (Questions listed below were presented in tabular format, with a 5-point Likert scale from 1 [strongly disagree] to 5 [strongly agree].)

The EAP presentation format...

1. Allowed you to adequately convey your thought process
2. Allowed adequate time for discussion of the patient’s subjective experience
3. Encouraged you to distill pertinent information in your presentation
4. Encouraged you to integrate information from the history, exam, and studies in developing an assessment and plan
5. Encouraged you to focus on your assessment and plan
6. Helped you learn from your own patients
7. Helped you learn from your peers
8. Is effective in advancing patient care
9. Is time-efficient
10. Is easy to use

*Section 5: EAP* (cont.)

Free Response 1

What do you like about the EAP format?

Free Response 2

What do you dislike about the EAP format?

*Section 6: Preference* (for those who chose option 3 in Section 3)

Which presentation format do you prefer? (the order of the following options was randomized)

▢ Subjective Objective Assessment Plan (SOAP) (1)

▢ Events Assessment Plan (EAP) (2)

▢ Neither, I do not have a preference (3)

▢ Other (please describe) (4)

Free Response 1

Why do you prefer the option you selected? Please note that we plan to use quotes from responses in this section for our paper.

*Section 7: Demographics*

What is your role?

▢ Medical student (1)

▢ Resident physician (2)

Display This Question: If What is your role? = Medical student (Med Stud Year)

What year are you?

▢ M3 (1)

▢ M4 (2)

Display This Question: If What is your role? = Resident physician (Resident Year)

What year are you?

▢ Intern (1)

▢ 2nd year resident (2)

▢ 3rd year resident (3)

▢ 4th year resident (4)

Display This Question: If What is your role? = Medical student (Med Student Age)

What is your age?

▢ under 21 years old (1)

▢ 21 - 24 years old (2)

▢ 25 - 29 years old (3)

▢ 30 - 34 years old (4)

▢ 35 - 39 years old (5)

▢ 40 years or older (6)

*Section 7: Demographics* (cont.)

Display This Question: If What is your role? = Resident physician (Resident Age)

What is your age?

▢ 25 - 29 years old (1)

▢ 30 - 34 years old (2)

▢ 35 - 39 years old (3)

▢ 40 years or older (4)

What gender do you identify with?

▢ Male (1)

▢ Female (2)

▢ Non-binary / third gender (3)

▢ Prefer not to say (4)

What is your race? (check all that apply)

▢ White (1)

▢ Black or African American (2) s

▢ Asian (3)

▢ Native Hawaiian or Pacific Islander (4)

▢ American Indian or Alaskan Native (5)

▢ Other (6)

▢ Prefer not to say (7)

Are you Hispanic or Latino?

▢ Yes (1)

▢ No (2)
